# Optimizing long chain-polyunsaturated fatty acid synthesis in salmonids by balancing dietary inputs

**DOI:** 10.1371/journal.pone.0205347

**Published:** 2018-10-10

**Authors:** Stefanie M. Colombo, Christopher C. Parrish, Manju P. A. Wijekoon

**Affiliations:** 1 Department of Animal Science and Aquaculture, Faculty of Agriculture, Dalhousie University, Truro, Nova Scotia, Canada; 2 Department of Ocean Sciences, Memorial University of Newfoundland, St. John’s, Newfoundland and Labrador, Canada; 3 Canadian Food Inspection Agency- Meat Hygiene Program, Guelph Ontario, Canada; University of Illinois, UNITED STATES

## Abstract

The increasing use of terrestrial plant lipids to replace of fish oil in commercial aquafeeds requires understanding synthesis and storage of long chain-polyunsaturated fatty acids (LC-PUFA) in farmed fish. Manipulation of dietary fatty acids may maximize tissue storage of LC-PUFA, through increased production and selective utilization. A data synthesis study was conducted to estimate optimal levels of fatty acids that may maximize the production and storage of LC-PUFA in the edible portion of salmonids. Data were compiled from four studies with Atlantic salmon, rainbow trout, and steelhead trout (total n = 180) which were fed diets containing different terrestrial-based oils to replace fish oil. LC-PUFA (%) were linearly correlated between diet and muscle tissue (p < 0.001; r^2^ > 44%), indicating proportional storage after consumption. The slope, or retention rate, was highest for docosahexaenoic acid (DHA) at 1.23, indicating that an additional 23% of DHA was stored in the muscle. Dietary saturated fatty acids were positively related to DHA stored in the muscle (p < 0.001; r^2^ = 22%), which may involve membrane structural requirements, as well as selective catabolism. DHA was found to be optimally stored with a dietary n-3: n-6 ratio of 1.03: 1. These new results provide a baseline of optimal dietary ratios that can be tested experimentally to determine the efficacy of balancing dietary fatty acids for maximum LC-PUFA storage.

## Introduction

The need to reduce fish meal (FM) and fish oil (FO) levels in aquafeeds has become critical with the growth of the aquaculture industry. This has been, and continues to be, a challenge because FM and FO provide unique nutrients that are required by finfish species, at varying levels depending on species and life stage. In particular, marine resources (i.e., FM and FO) contain significant levels of long-chain polyunsaturated fatty acids (LC-PUFA; ≥ 20 carbons in length), namely the n-3 and n-6 LC-PUFA: eicosapentaenoic acid (EPA; 20:5n-3), docosahexaenoic acid (DHA; 22:6n-3), and arachidonic acid (20:4n-6). These LC-PUFA play numerous physiologically important roles essential to health of all vertebrates [1] and are primarily produced and abundant in marine ecosystems [2]. Although vertebrates have some ability to synthesise LC-PUFA from the C_18_ precursors linoleic acid (LNA, 18:2n-6) and α-linolenic acid (ALA, 18:3n-3), dietary supply of these LC-PUFA is still required to meet physiological demands. Fish are the primary source of n-3 LC-PUFA for humans [3], and this has prompted interest in LC-PUFA metabolism in fish, with biosynthesis being one of the most targeted pathways under investigation.

The levels of FM and FO in commercial aquafeed formulations have been reduced due to replacement by terrestrial plant meals and oils [4, 5] (Tacon and Metian 2015; Ytrestøyl et al. 2015). However, because terrestrial plants do not produce EPA and DHA [2], some level of FM and FO are needed to meet finfish LC-PUFA requirements [6]. For species that primarily store lipid in flesh (e.g., salmonids), it has been extensively reported that the fatty acid (FA) content of fillets is highly reflective of the diet FA content. As such, using high levels of terrestrial ingredients to replace FM and FO cannot be accomplished without compromising product quality through reduced flesh LC-PUFA content [7]. In fact, this practice has resulted in significant increases in the amounts of ALA and LNA stored in Atlantic salmon (*Salmo salar*) fillets, and subsequent decreases in EPA and DHA over the past decade [8]. Consequently, the nutritional value of the final product is compromised.

Understanding the genetic basis of LC-PUFA synthesis in finfish has become increasingly important in the context of aquaculture. This research has led to new discoveries to improve utilization, synthesis, and storage of the LC-PUFA. In the long term, this may enable very minimal inclusion of FM and FO, or perhaps eliminate dietary inclusion all together. Part of this bigger picture includes understanding the relationship between dietary LC-PUFA input and output, i.e., the rate and quantity of LC-PUFA synthesis and production. Dietary ratios among the LC-PUFA, such as DHA/EPA and EPA/ARA, are also known to influence the phenotypic response, including growth, lipid metabolism, and LC-PUFA synthesis [9, 10, 11]. Defining, and quantifying, optimal dietary ratios to achieve high muscle tissue storage levels of LC-PUFA would be helpful from a production and consumption standpoint. While many single-experiment studies have evaluated tissue FA content in response to dietary FA changes, a comprehensive examination of several studies has not been conducted to yield more general conclusions regarding dietary FA optimization.

We took a data synthesis approach to understand the relationship between FA input and LC-PUFA output by compiling data from two experiments with Atlantic salmon [10, 12], one experiment with rainbow trout (*Oncorhynchus mykiss)* [13], and one with steelhead trout (*Oncorhynchus mykiss)* [14]. In each experiment, the diets replaced some level of FO with various terrestrial lipid sources (e.g., camelina, canola, flaxseed, and sunflower oil). The objective of our data synthesis was to broadly understand the utilization of dietary FA to optimize the synthesis and storage of LC-PUFA in a commercially-relevant teleost family, and to identify ratios and levels of LC-PUFA inputs to yield optimal output, specifically looking at the plasticity of the composition of the edible proportion of the fish.

## Materials and methods

### Data acquisition

Diet and muscle tissue FA data were collected from four different studies: Atlantic salmon experiment 1 [12], Atlantic salmon experiment 2 [10], rainbow trout [13], and steelhead trout [14]. Complete experimental details can be found in the original publications, including source of fish, source of dietary ingredients, approved animal care protocol numbers, growth performance, feed intake, proximate composition of diets and muscle tissue, lipid extraction and fatty acid analytical methods, and complete dietary and tissue FA profiles. All experimental procedures involving fish were performed in accordance with the guidelines approved by the Institutional Animal Care and Use Committees at Dalhousie University and Memorial University of Newfoundland. A brief summary of the design and test conditions employed in the four studies follows.

#### Atlantic salmon (Experiment 1)

The experimental treatments were as follows: a control diet with fish oil (FO) at 14% of the diet; a full replacement of FO with camelina oil (CO); a full replacement of FO with CO and addition of solvent extracted FM (SEFM); a full replacement of FO with CO and 10% inclusion of camelina meal; and a full replacement of FO with CO plus SEFM plus 10% inclusion of CM. The addition of both solvent extracted FM and camelina meal further removed all dietary LC-PUFA. The experiment was conducted in seawater at 14°C (233.5 ± 46 g fish^-1^ mean initial weight; 26.5 ± 1.8 cm mean initial length) in a flow-through system at the Department of Ocean Sciences (Memorial University of Newfoundland, St. John’s, Newfoundland and Labrador, Canada) for 16 weeks. The smolts were randomly distributed (300 total) into experimental tanks (500 L), with triplicate tanks per treatment and 50 fish per tank. Fish were fed to apparent satiation twice daily and feed intake was recorded. Mortalities were weighed and recorded throughout the trial. After 16 weeks, skinless, dorsal muscle tissue was sampled from three fish per tank for subsequent lipid and fatty acid analysis. This study was previously published in Hixson et al. [12].

#### Atlantic salmon (Experiment 2)

The experimental treatments replaced FO with either CO or canola oil (10 treatments). The control diet contained FO (24% of the diet). The CO diets included: a high level of inclusion (23% of the diet, 1% FO), medium inclusion (21.5% of the diet, 2.5% FO), and low inclusion (19% of the diet, 5% FO). The canola oil diets included: a high canola oil inclusion (23% of the diet), medium inclusion (21.5% of the diet), low inclusion (19% of the diet). The following three diets included CO at increasing levels, with the addition of poultry fat at a constant level (at 14% of the diet). These diets consisted of a high CO inclusion (8.5% of the diet) plus addition of poultry fat (14%), a medium CO inclusion (7.1%) plus poultry fat (14%), and a low CO inclusion (4.6%) plus poultry fat (14%). The experiment was conducted in seawater at 14°C (256 ± 28 g fish ^-1^ mean initial weight ± SD; 29 ± 1.1 cm mean initial length) in a flow through system at the Department of Ocean Sciences for 16 weeks. The smolts (1500) were randomly distributed into 30 experimental tanks (620 L), each tank with 50 fish. Fish were fed to apparent satiation, and feed intake was recorded. Mortalities were weighed and recorded throughout the trial. After 16 weeks, skinless, dorsal muscle tissue was sampled from three fish per tank for subsequent lipid and fatty acid analysis. This study was previously published in Hixson et al. [10].

#### Rainbow trout

The experimental diets were as follows: a control diet with FO at 17.5% of the diet, a 50% replacement of FO with CO, and a full replacement of FO with CO. An experiment was conducted with juvenile rainbow trout in freshwater at 14°C (44.9 ± 10 g fish^-1^ mean initial weight; 15.7 ± 1.2 cm mean initial length) in a flow through system at the Faculty of Agriculture, Dalhousie University (Truro, Nova Scotia, Canada) for 12 weeks. The fish were randomly distributed (837 total) into experimental tanks (200 L capacity), with triplicate tanks per treatment and 93 fish per tank. The fish were fed to apparent satiation twice daily and feed intake was recorded. After 12 weeks, skinless, dorsal muscle tissue was sampled from three fish per tank for subsequent lipid and fatty acid analysis. This study was previously published in Hixson et al. [13].

#### Steelhead trout

The experimental treatments were as follows: a control diet with FO (29% lipid), a full replacement of FO with sunflower oil (26% lipid), and a full replacement of FO with flaxseed oil (22% lipid). The experiment was conducted in seawater at 14.5°C (120 g fish ^-1^ mean initial weight) in a flow through system at the Department of Ocean Sciences for 12 weeks. Fifty-five fish were haphazardly picked and distributed to each experimental tank (6 × 6000 L tanks). Muscle samples for lipid class and fatty acid analysis were obtained from the left epaxial region of the fish caudo-dorsal to the pectoral and ventral to the anterior base of the dorsal fin. Fish were fed to apparent satiation, and feed intake was recorded. This study was previously published in Wijekoon et al. [14].

### Data analysis

A selection of relevant FA were chosen from each experiment to determine relationships between dietary and muscle tissue FA. Dietary FA included: ALA, LNA, oleic acid (OA; 18:1n-9), EPA, DHA, and ARA, sum of saturated FA (SFA), sum of monounsaturated FA (MUFA), and the ratio of ALA: LNA. The LC-PUFA were the main focus of the muscle tissue (EPA, DHA, and ARA). Diet and muscle tissue FA from each study are summarized in Table 1; only the control diet (FO) and treatments with the highest level of FO replacement were summarized in this Table.

**Table 1:** Summary of diet and tissue FA (% total fatty acids and ratios) of interest from 4 different studies. Atlantic salmon and rainbow trout were fed diets containing fish oil (FO), camelina oil (CO)^1^ and canola oil (CA)^1^, and steelhead trout were fed sunflower oil (SF) or flaxseed oil (FX) compared to fish oil (FO).

	Atlantic Salmon 1		Atlantic Salmon 2			Rainbow Trout		Steelhead Trout				Summary	
**Diet**	FO	CO	FO	CO	CA	FO	CO	FO	SF	FX	Max	Min	Ratio
ALA	0.8	24.2	0.9	26.5	7.1	1.1	20.2	1.1	1.6	37.4	37.4	0.8	46.8
LNA	5.6	19.0	13.0	19.3	19.5	7.8	20.5	7.2	23.0	15.0	23	5.6	4.1
ALA:LNA	0.1	1.3	0.1	1.4	0.4	0.1	1.0	0.2	0.1	2.5	2.5	0.1	25.0
LNA:ALA	7.0	0.8	14.4	0.7	2.8	7.1	1.0	6.5	14.4	0.4	14.4	0.4	36.0
OA	8.5	21.2	6.6	17.2	54.4	10.6	19.0	8.1	32.4	11.3	54.4	6.6	8.2
EPA	15.5	0.6	5.7	0.4	0.4	11.8	2.2	16.8	5.0	4.5	16.8	0.4	42.0
ARA	0.7	>0.1	0.3	0.1	n.d	0.7	0.2	1.1	0.3	0.3	1.1	0.1	11.0
DHA	7.9	0.9	4.9	0.5	0.4	6.8	2.8	8.8	4.4	4.2	8.8	0.4	22.0
SFA	27.7	11.6	19.2	12.5	10.2	37.1	17.7	26.6	16.0	14.3	37.1	10.2	3.6
MUFA	32.5	40.7	56.1	37.3	62.3	28.7	33.9	25.4	45.9	20.8	62.3	20.8	3.0
**Tissue**													
ALA	0.9 ± 0.1	12.6 ± 1.0	0.9 ± 0.1	5.3 ± 0.8	2.8 ± 0.3	5.7 ± 0.3	14.5 ± 0.4	0.5 ± 0.1	2.4 ± 0.5	8.9 ± 1.8	14.5	0.5	29.0
LNA	5.8 ± 0.8	14.0 ± 0.6	7.7 ± 1.0	14.6 ± 0.3	13.5 ± 1.2	1.2 ± 0.1	12.6 ± 0.4	5.6 ± 0.4	11.4 ± 1.7	9.4 ± 2.2	14.6	1.2	12.2
ALA:LNA	0.2 ± 0.01	0.9 ± 0.03	0.1 ± 0.01	0.9 ± 0.03	0.2 ± 0.01	4.7 ± 0.3	1.1 ± 0.02	0.1 ± 0.02	0.2 ± 0.03	1.0 ± 0.2	4.7	0.1	47.0
LNA:ALA	6.3 ± 0.5	1.1 ± 0.04	8.8 ± 0.3	1.2 ± 0.1	4.9 ± 0.2	0.2 ± 0.01	0.9 ± 0.01	10.2 ± 1.1	4.8 ± 0.8	1.1 ± 0.5	10.2	0.2	51.0
EPA	9.0 ± 0.7	2.1 ± 0.2	3.2 ± 0.3	2.0 ± 0.3	1.3 ± 0.1	9.5 ± 0.4	2.3 ± 0.3	9.6 ± 1.0	4.9 ± 1.4	6.1 ± 1.0	9.6	1.3	7.4
ARA	0.8 ± 0.1	0.4 ± 0.5	0.4 ± 0.1	0.5 ± 0.1	0.7 ± 0.2	0.8 ± 0.1	2.0 ± 0.1	1.4 ± 0.1	1.6 ± 0.2	1.1 ± 0.1	2.0	0.4	5.0
DHA	13.3 ± 2.9	3.9 ± 0.1	7.7 ± 1.0	6.5 ± 0.8	4.9 ± 0.7	13.4 ± 0.9	6.6 ± 1.2	27.4 ± 1.7	20.1 ± 4.6	23.2 ± 4.1	27.4	3.6	7.0
n-3: n-6^2^	3.9 ± 0.8	1.5 ± 0.1	1.6 ± 0.1	1.6 ± 0.1	0.7 ± 0.1	3.9 ± 0.3	1.6 ± 0.1	5.0 ± 0.4	2.1 ± 0.1	3.3 ± 0.1	5.0	0.7	7.1
n-6: n-3^2^	0.3 ± 0.01	0.7 ± 0.1	0.2 ± 0.01	0.6 ± 0.02	1.3 ± 0.1	0.3 ± 0.01	0.6 ± 0.1	0.2 ± 0.02	0.5 ± 0.15	0.3 ± 0.08	1.3	0.2	6.5

^1^These treatments represent the highest level of FO (and FM) replacement in the experiment.

#### Regression analysis

Relationships among diet and muscle tissue FA were analyzed using linear regression in Sigma Plot (version 13.0; Systat Software Inc., San Jose, California, USA). All diet FA (independent variable) were related to muscle tissue LC-PUFA, and LC-PUFA in the diet were also related to LC-PUFA in the muscle tissue. Dietary total lipid (%) was also related to muscle tissue LC-PUFA, both as proportion (%) and quantitative (μg/g). Species was used as a categorical variable to determine if there was a difference in linear regression among salmon, rainbow trout, and steelhead trout. A dynamic curve-fit function was used when linear regression did not provide an adequate description of the data. Segmented fits were used when there appeared to be opposing linear relationships within the data. On the x-axis (dietary FA), where the fitted functions intersect is defined as the breakpoint [15], and represents a value where the relationship between dietary FA level and muscle tissue FA level changes. The value of the breakpoint is estimated in the analysis. When there is only one breakpoint at x = T, the model is written as follows: y = a1 +b1x < T; y = a2 +b2x > T.

In order to test the strength of our models to predict tissue LC-PUFA based on diet FA, we collected diet and tissue FA data from the literature from studies that tested various feeds in farmed salmonids (see S1 File). We used our linear models with the published diet FA input in order to predict the tissue LC-PUFA. To validate the effectiveness of the model, we regressed the observed tissue LC-PUFA values (from published data, y-axis) against the predicted values (from our model, x-axis). We also compared the observed and predicted LC-PUFA means using a *t*-test.

#### ANOVA

Tissue-to-diet concentration (μg g^-1^) ratios for ALA, LNA, EPA, DHA, and ARA were calculated for Atlantic salmon and rainbow trout, as quantitative FA data were available from those studies only. A two-way ANOVA was used to determine if species (rainbow trout or salmon) or diet (FO or CO) were factors that determined the tissue-to-diet ratio (response variable). Experiment as a factor was tested separately in a one-way ANOVA, as the previous design would have been unbalanced with this factor included. The models were run using Minitab Statistical Software (Minitab 17.3, State College Pennsylvania, USA).

#### Multivariate statistics

All multivariate analyses were conducted using PRIMER-E (Plymouth Routines in Multivariate Ecological Research; PRIMER-E Ltd, version 7.0.10, Ivybridge, UK). The data set was square-root transformed prior to analysis in PRIMER. Principal coordinates analysis (PCO) was used to visualize and quantify patterns observed among muscle tissue LC-PUFA. To evaluate the significance of dietary LC-PUFA and species separation observed in the PCO, we used a one-way permutational multivariate analysis of variance (PERMANOVA). In order to categorize the dietary FA values as explanatory factors, they were divided into four quartiles (using the descriptive statistics function in Minitab), with a low dietary FA value assigned to 1 and a high dietary FA value assigned to 4. Therefore, for each fish, it was assigned a number between 1 and 4, depending on its diet. Similarity in Percentages (SIMPER) was used to relate individual muscle tissue LC-PUFA (response variables) with dietary FA (categorical variable). The SIMPER analysis then determines differences between dietary FA quartiles based on muscle tissue LC-PUFA response and determines the muscle tissue FA that is most contributing to this difference. The non-metric Bray-Curtis dissimilarity statistic was used to quantify the compositional dissimilarity between samples in the PCO and SIMPER [16]. This test delivers robust and reliable dissimilarity results and is one of the most commonly used metrics to explore relationships in ecology, environmental sciences and related fields [17].

## Results

### Diet and muscle tissue FA relationship

**Fig 1:**
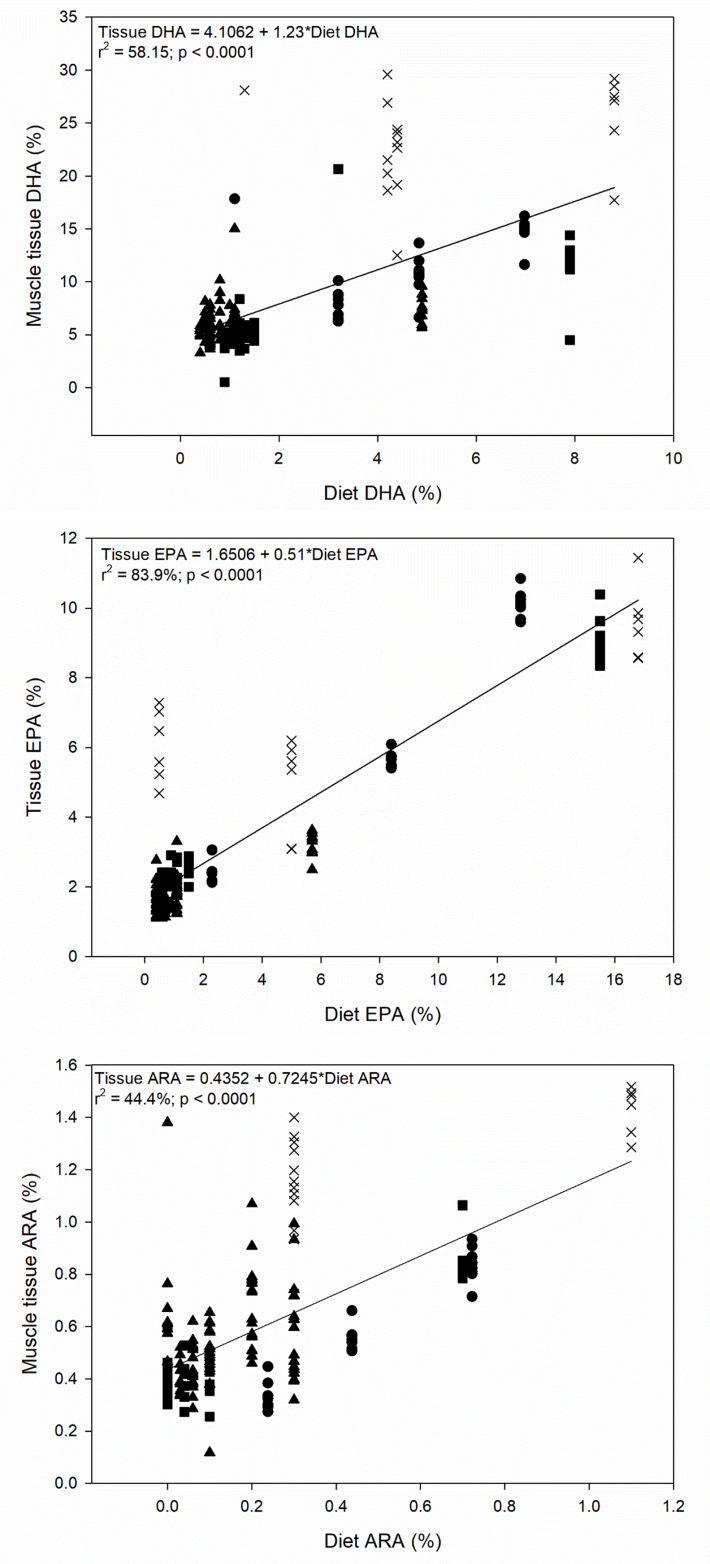
Linear regression (± SE = standard error of estimate) of diet LC-PUFA vs. muscle tissue LC-PUFA for Atlantic salmon 1 (■) and 2 (▲), rainbow trout (●), and steelhead trout (×) for a) DHA, b) EPA, and c) ARA.

**Fig 2:**
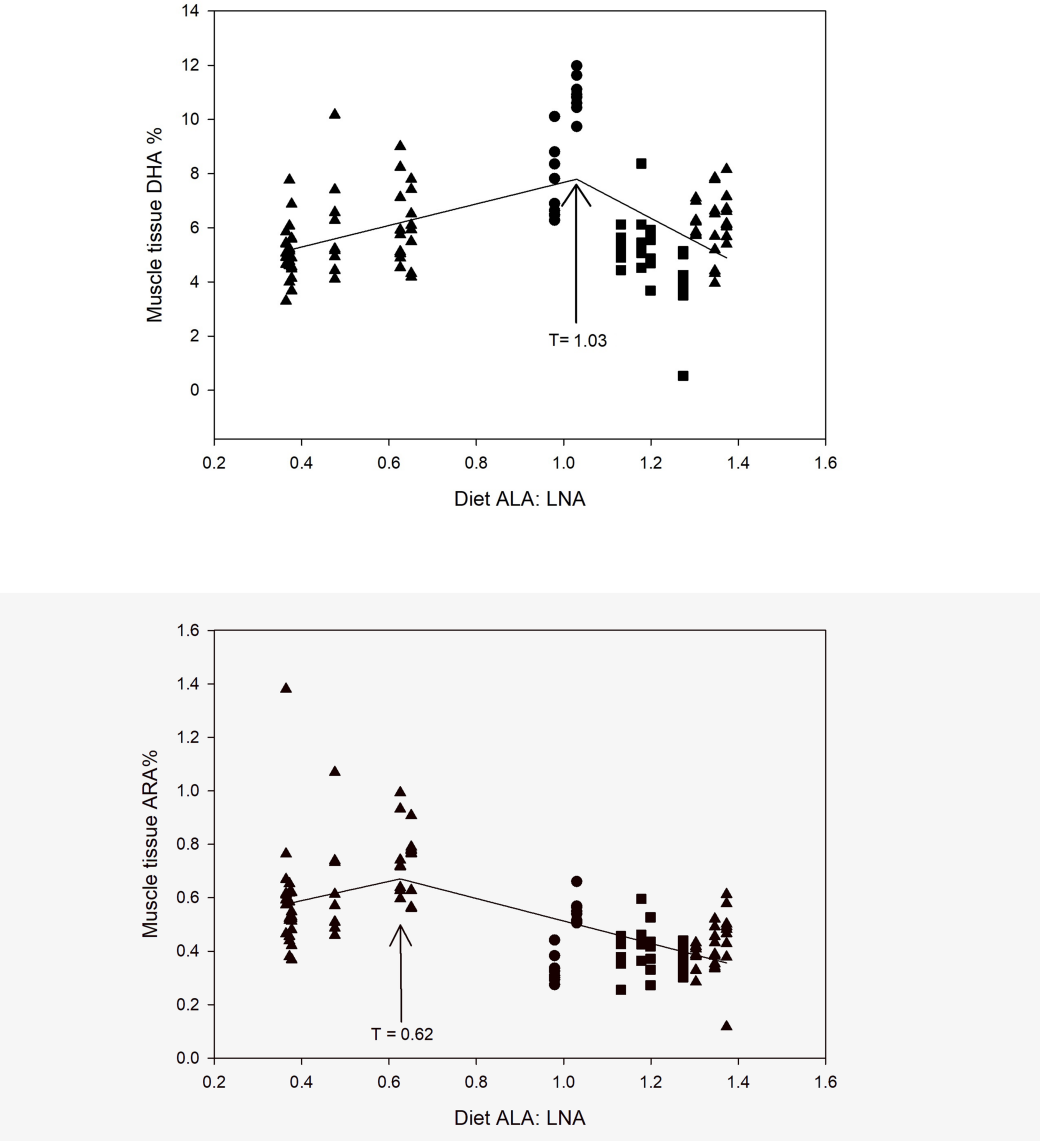
Segmented relationship between dietary ALA: LNA ratio and the proportion LC-PUFA in the muscle tissue of Atlantic salmon 1 (■) and 2 (▲), and rainbow trout (●) for: a) DHA: DHA = 2.41+(6.74*ALA:LNA) ≤ T, DHA = 17.8-(9.32*ALA:LNA) ≥ T; and b) ARA: ARA = 0.35+(0.60*ALA:LNA) ≤ T; ARA = 0.36-(0.17*ALA:LNA) ≥ T.

Dietary ALA, LNA, and total MUFA all showed negative linear relationships with muscle LC-PUFA (Table 2). Dietary LC-PUFA (DHA, EPA, and ARA) all showed positive linear relationships with the same LC-PUFA in the muscle tissue (p < 0.001; r^2^ = 44–84%; Table 2 and Fig 1A, 1B and 1C). Total SFA in the diet and ARA in the diet were both positively related to DHA in the tissue. A segmented regression showed that the highest muscle tissue DHA levels when the dietary ratio of ALA: LNA was T = 1.03 ± 1.90 (SE, standard error of the estimate; Fig 2A), and the highest muscle tissue ARA levels were found at T = 0.62 ± 0.14 (Fig 2B). ALA in the muscle tissue increases with higher dietary ratio of ALA: LNA (S1 Fig), while LNA approaches an exponential maximum when the ALA: LNA ratio reaches approximately 0.5 (S2 Fig). The LNA: ALA ratio in the diet showed a less distinct, yet negative relationship with LNA stored in the muscle tissue (slope = 0.37; p < 0.01; r^2^ = 0.05). Another segmented regression showed that muscle tissue DHA significantly decreased with higher levels of OA in the diet, up to T = 19.8 ± 4.97% OA (S3 Fig); however, beyond that point, the linear relationship was not significant (p = 0.99), but the slope was < 0.001. Species was a significant categorical factor in all relationships among diet and muscle tissue FA.

**Table 2:** Linear regression results among diet and muscle tissue fatty acid relationships (± standard error of estimate, SE).

Independent (x)	Dependent (y)	Linear Equation	p-value	r^2^	SE
Diet ALA	Tissue DHA	Tissue DHA = 10.66 - (0.13 * Diet ALA)	0.003	0.05	5.95
Diet ALA	Tissue EPA	Tissue EPA = 4.89 - (0.10 * Diet ALA)	<0.001	0.02	2.56
Diet ALA	Tissue ARA	Tissue ARA = 0.76 - (0.01 * Diet ALA)	<0.001	0.15	0.27
Diet LNA	Tissue DHA	Tissue DHA = 20.06 - (0.69 * Diet LNA)	<0.001	0.26	5.26
Diet LNA	Tissue EPA	Tissue EPA = 11.63 - (0.51 * Diet LNA)	<0.001	0.02	1.62
Diet LNA	Tissue ARA	Tissue ARA = 0.76 - (0.01 * Diet LNA)	<0.001	0.15	0.26
Diet SFA	Tissue DHA	Tissue DHA = 1.43 + (0.42 * Diet SFA)	<0.001	0.21	5.42
Diet SFA	Tissue EPA	Tissue EPA = -1.61 + (0.29 * Diet SFA)	<0.001	0.47	2.01
Diet SFA	Tissue ARA	Tissue ARA = 0.24 + (0.02 * Diet SFA)	<0.001	0.23	0.25
Diet MUFA	Tissue DHA	Tissue DHA = 23.28 - (0.34 * Diet MUFA)	<0.001	0.36	4.87
Diet MUFA	Tissue EPA	Tissue EPA = 10.78 - (0.17 * Diet MUFA)	<0.001	0.45	2.05
Diet MUFA	Tissue ARA	Tissue ARA = 0.99 - (0.01 * Diet MUFA)	<0.001	0.12	0.27
Diet ARA	Tissue DHA	Tissue DHA = 4.66 + (17.47 * Diet ARA)	<0.001	0.57	3.99
Diet ARA	Tissue EPA	Tissue EPA = 1.24 + (9.29 * Diet ARA)	<0.001	0.79	1.28
Diet DHA	Tissue ARA	Tissue ARA = 0.45+ (0.06 * Diet DHA)	<0.001	0.31	0.24

The relationship between total lipid and muscle LC-PUFA was not significant. However, the relationship between total lipid and the amount (μg/g) of LC-PUFA in the muscle was significant (p < 0.001). Steelhead trout was excluded from this dataset because quantitative amounts of FA were not available. As a result, there was a gap between 18.5% - 27.3% total lipid; therefore, more data are needed to confirm this relationship.

We tested the strength of the linear models by using published diet FA data to predict the muscle tissue LC-PUFA levels, and compared the model values with the actual values using regression and *t*-tests. The LC-PUFA diet *vs* tissue models were the strongest, showing significant linear regressions of observed *vs* predicted LC-PUFA tissue values and *t*-tests comparing the means of observed *vs* predicted values (Table 3). Table 3 shows the linear relationships that significantly predicted the observed values from published data. The remaining regression models tested were not strong predictors of tissue LC-PUFA according to our results from testing the observed *vs* predicted values, and are shown in S1 Table. The data collected from the literature to test the models is available in the S1 File.

**Table 3:** Results from model testing based on observed (from published data) *vs* predicted (model) tissue LC-PUFA values, showing models with the strongest explanatory values. To validate the effectiveness of the model, we regressed the observed tissue LC-PUFA values (from published data) against the predicted values (from our model). We also compared the observed and predicted LC-PUFA means using a *t*-test.

Model		Slope	r^2^	p-value	T-stat	p-value
Diet	Tissue					
DHA	DHA	0.74	35.5	<0.001	-0.73	0.463
EPA	EPA	1.04	73.5	<0.001	-0.60	0.549
ARA	ARA	1.02	32.3	<0.001	-2.23	0.027
ALA	DHA	1.36	13.9	<0.001	0.20	0.845
ALA	EPA	0.83	14.7	<0.001	-0.04	0.968
MUFA	EPA	0.25	5.2	0.028	-0.96	0.337
ARA	DHA	0.33	10.7	0.001	-0.69	0.488
ARA	EPA	0.56	47.8	<0.001	-1.34	0.182

### Tissue to diet ratios

**Fig 3:**
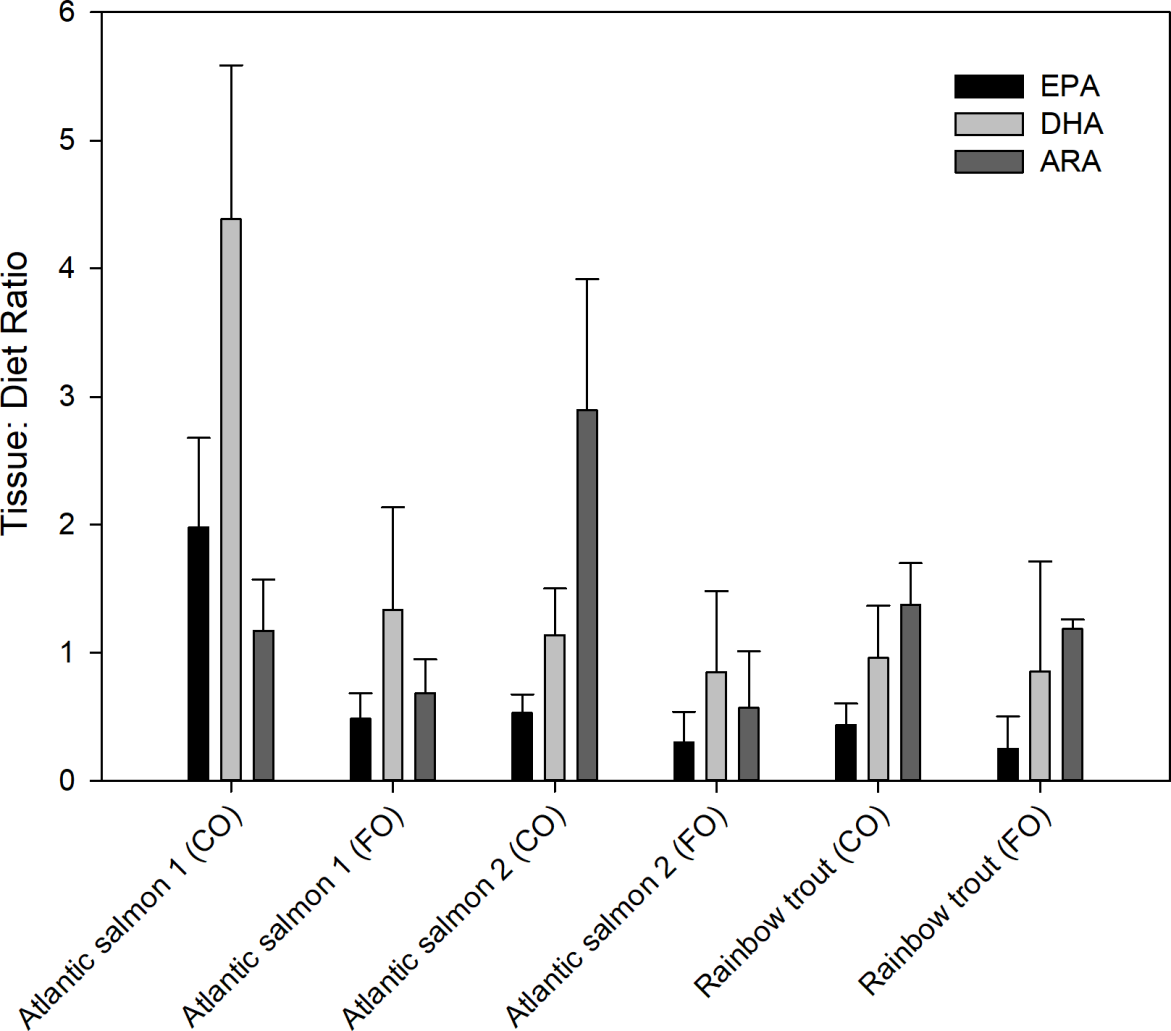
Tissue-to-diet concentration (μg g^-1^) ratio for EPA, DHA, and ARA in Atlantic salmon and rainbow trout.

The amount of LC-PUFA in the muscle tissue (μg g^-1^) was compared to the amount supplied in the diet (μg g^-1^) for Atlantic salmon and Rainbow trout (Fig 3). The tissue-to-diet ratio was higher in fish fed CO diets compared to those fed a FO-based diet. However, there was no agreement on which LC-PUFA showed the highest tissue-to-diet ratio when comparing species in CO treatments, although EPA consistently showed the lowest ratio among species. Considering EPA, DHA, and ARA separately as response variables, a two-way ANOVA determined that species (salmon or trout) and diet (FO or CO) were significant factors that determined the tissue-to-diet ratio for EPA and DHA (p < 0.01 for both factors in each model). For ARA, diet was a significant factor (p < 0.001), but species was not different (p = 0.61). A one-way ANOVA determined that experiment was a significant factor for EPA and DHA tissue-to-diet ratios (p<0.001), but not for ARA (p = 0.07).

**Fig 4:**
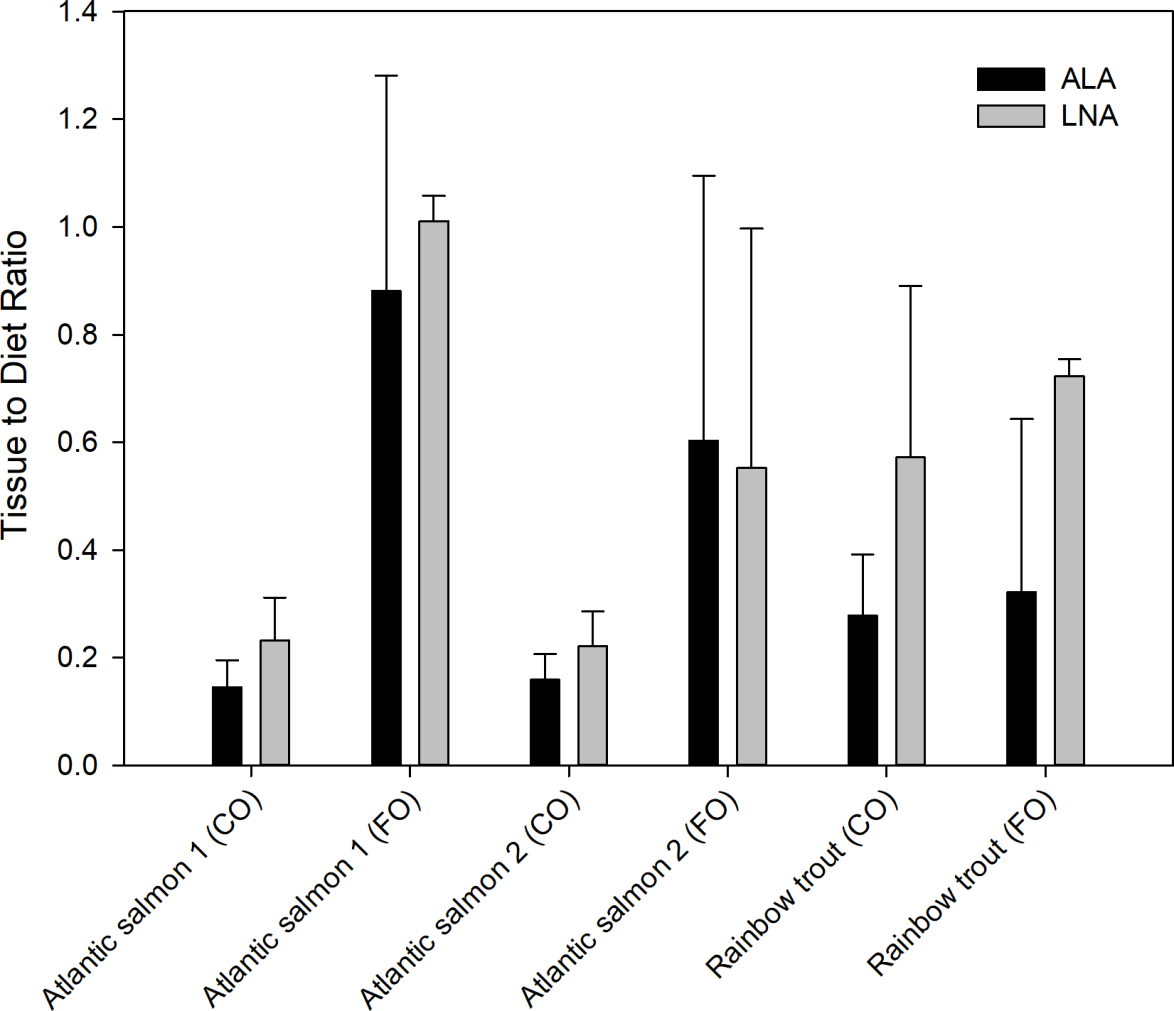
Tissue-to-diet concentration (μg g^-1^) ratio for ALA and LNA in Atlantic salmon and rainbow trout.

The tissue-to-diet ratios for ALA and LNA (Fig 4) were consistently lower than the LC-PUFA. Fish fed the CO diets showed lower tissue-to-diet ratios than those fed FO diets, regardless of treatment. Tissue-to-diet ratios for both ALA and LNA were different between CO and FO treatments (p<0.001), but species (ALA p = 0.14; LNA p = 0.06) and experiment (ALA p = 0.87; LNA p = 0.87) were not significant factors for either FA.

### Multivariate analysis

**Fig 5:**
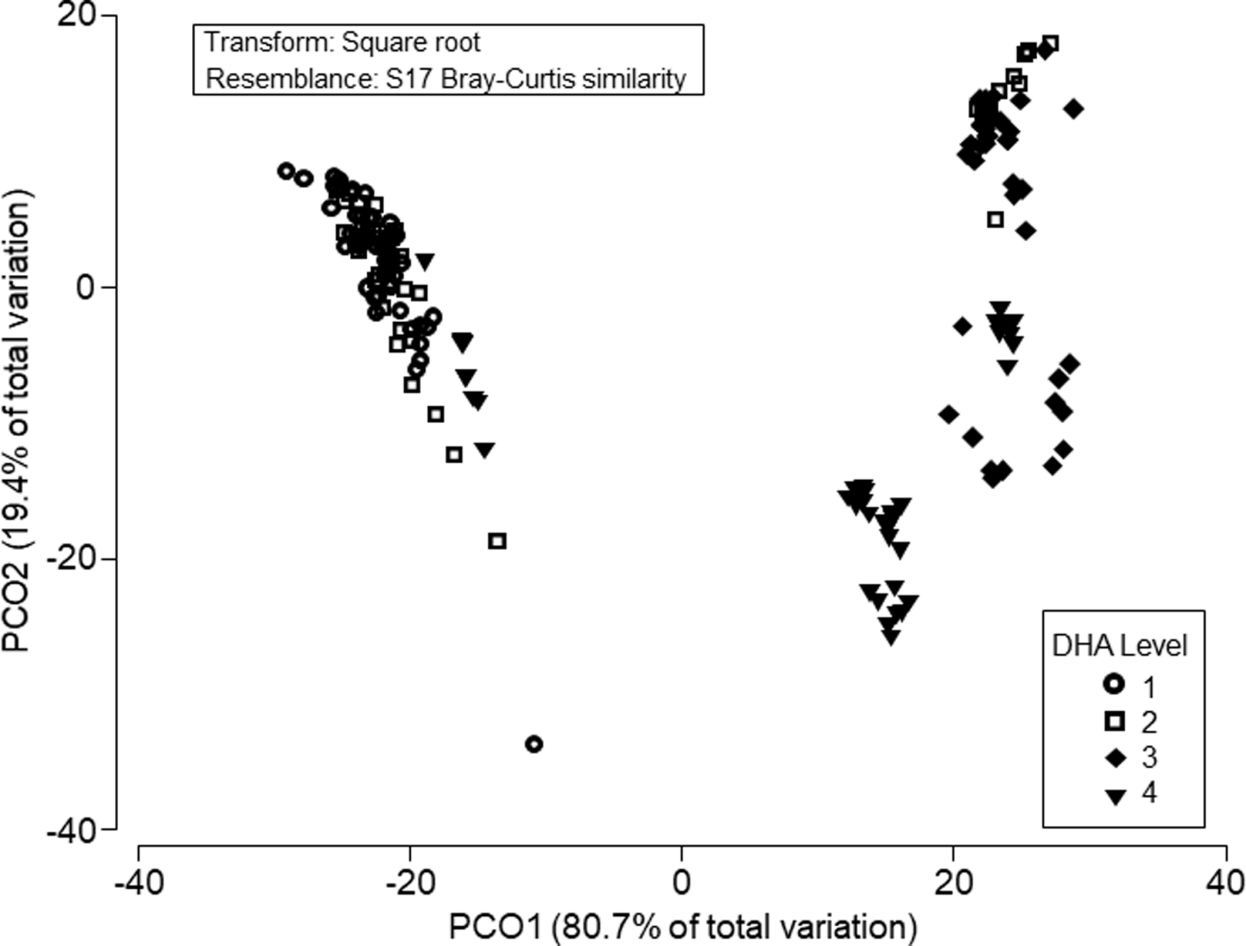
Principal coordinates ordination of data from all four experiments. Data points represent the LC-PUFA content from each individual and are categorized by dietary DHA level, with 1 being low, 4 is high. Diet DHA was not significant factor explaining the separation in the plot, however when nested within the species factor, DHA was significant (pseudo F = 192.63; p = 0.001.

In the PCO, PCO1 explained 80.7% of the variation and PCO2 explained 19.4% of the variation (Fig 5). A separation by species across PCO1 was evident, with salmon (experiment 2) on the negative side of PCO1 and salmon (experiment 1) slightly more negative than rainbow trout and steelhead trout. Diet FA appeared to be separated across PCO2. Using PERMANOVA, we found that diet DHA alone was not a significant factor in determining muscle tissue LC-PUFA, but when nested within species, it was significant (pseudo F = 192.63; p = 0.001). We found the same result for diet ALA when nested within species (pseudo F = 200. 1; p = 0.001).

Using SIMPER, we found that when fish were divided into four quartiles based on their diet DHA level, groups 1 (lowest diet DHA) and 3 were the most different (44% dissimilar), that is, showed the greatest difference in their tissue LC-PUFA content. For all group comparisons, SIMPER determined (Bray-Curtis dissimilarity statistic) that LNA was the tissue FA that contributed the most difference between all groups (31–44%). When the fish were divided into four quartiles based on diet ALA level, groups 2 (lowest diet ALA) and 3 were the most different (47% dissimilar). For all group comparisons, LNA again was the tissue FA that contributed the most difference between all groups (31–47%).

## Discussion

Retention of LC-PUFA in fillets has been a research concern since it has become necessary to reduce the level of FO and FM in aquafeeds. Providing a minimum level of LC-PUFA may be adequate to meet basic requirements for fish; however, levels stored in fillet tissue have decreased over time. It has been documented that EPA and DHA have lowered in commercial farmed-raised Atlantic salmon fillets [8]. In order to summarize our previous studies to identify a starting place for subsequent research, we compiled data from four previous experiments to pinpoint optimal levels and ratios of dietary FA that encourage maximum production and yield the highest levels of LC-PUFA in fillets, when feeding diets that are low or devoid in FO. While our study is limited to those compiled from these experiments, these single studies may be representative of other similar experiments that have replaced fish oil in diets for salmonids, so our conclusions could be broadly applicable. However, pooling our data from different species did introduce some variation, and was a significant factor in our regression and multivariate models. Clearly species is a defining factor in the muscle LC-PUFA content between salmon and trout. This could be attributed to lipid and fatty acid metabolism and total dietary lipid levels formulated on a per species basis. Some of the variation is also caused by differences in experimental methods among trials: differences in total lipid in the diet, different diet compositions, different feeding durations, and different rearing temperatures. However, we found patterns in the data despite species differences, which helps make broad inferences on the relationship between diet and tissue composition in a large family of teleosts. While not all relationships that we report in this study are strong explanatory models, this was part of the exploratory process, to determine which relationships could be the strongest predictors of LC-PUFA muscle tissue storage. This can then be used as a starting place for experimental studies *in vivo*. Using muscle tissue response data from limited dietary lipid sources to investigate dietary FA and LC-PUFA storage we are able to investigate optimal dietary FA levels and ratios. Our study utilized a novel method which draws new, broad conclusions on optimal levels of dietary FA to promote synthesis and storage of LC-PUFA.

### Direct diet to muscle tissue storage

LC-PUFA are highly retained and conserved from diet to tissue in fish [18, 19, 20]. The LC-PUFA we investigated showed a linear relationship between the level in the diet and that stored in the muscle. It is interesting to note the slope of the relationship, which represents the rate of retention from diet to muscle tissue. For every one unit of DHA provided in the diet, 1.23 units are stored in the muscle tissue (see Fig 1B). This suggests that an additional 23% of DHA is stored beyond that which was provided in the diet. This value is suggestive of DHA synthesis as it is remarkably close to proportions synthesized in Atlantic salmon (25%) [21] and rainbow trout (27%) [13], which were determined using distinctly different methods, the fatty acid mass balance method [22] and compound specific stable isotope analysis [23].

In salmonids, muscle FA is highly reflective of diet FA; however, not all dietary DHA is stored directly in the muscle tissue. Nonetheless, our data suggest that of the available DHA that can be stored in the muscle, more than the available diet proportion is found there, which likely is from de novo synthesis. Tracer FA studies can accurately compartmentalize all dietary DHA into various tissues, on a quantitative basis, to quantify exactly where DHA is stored or metabolized, such as compound specific stable isotope analysis and the fatty acid mass balance method (as mentioned previously), as well as ^14^C labelling of ALA and/or LNA (e.g., [24, 25]).

For EPA or ARA, the value of the slope was less than one, suggesting that less than the entire proportion of diet EPA and ARA was reflected in the muscle tissue, and may have been stored or sequestered for other purposes. Only about half of the EPA that was consumed was stored in the muscle tissue. This value is also reasonable though, considering that EPA is the n-3 precursor for eicosanoid synthesis, and some of the EPA may be also be modified toward DHA production. This does not necessarily suggest that EPA and ARA are taken up only from the diets; it means that less EPA and ARA are stored in muscle than is supplied in the diet. Because both EPA and ARA are sequestered for other purposes, there could be some synthesis occurring, even if not all dietary EPA and ARA are entirely stored in the muscle. However, to know this for sure would require a method such as compound specific isotope analysis to know the origin of the FA. Further, EPA is also known to be more dispensable [26, 27, 28], and is preferentially catabolized in salmonids, if dietary requirements have been met [29]. ARA showed slightly higher retention than EPA (72%); yet was still not completely stored from the diet. ARA has been found to be the preferred substrate for eicosanoid production (rather than EPA) [30, 31], which could explain why the retention was lower than DHA. High retention of DHA and ARA highlight their importance in salmonids, supporting that DHA (and ARA) are the primary drivers of LC-PUFA essentiality in some fish species [9, 26].

The significant intercept of the regression between diet and muscle tissue is also worth noting. The value may indicate minimal levels in muscle, i.e. what is required in membranes. For example, considering that 10% of total lipid in muscle tissue is phospholipid in salmonids (based on the phospholipid levels in the salmon in the experiments used in this study) [10, 12, 13], then 4% DHA would equate to approximately 40% DHA if it was completely esterified in membrane phospholipids, which is a reasonable upper limit in farmed salmon, farmed trout and other marine fish species [32, 33]. This is also the case for ARA in phospholipid, where 0.4% would equate to 4% ARA, which again would be a reasonable upper limit in fish phospholipids, given the high proportions found in phosphatidylinositol [32, 33].

The total lipid values range from 14–29% of the diet. This should minimally affect our overall conclusions because we evaluated fatty acid proportions (which are independent of total lipid) and relative changes were assessed, which is an appropriate method to compare among experiments with different dietary total lipids. Comparing total lipid with muscle LC-PUFA proportions did not reveal any significant relationships. However, comparing dietary lipid with quantitative amounts of LC-PUFA in the muscle, indicated there may be relationship, but more samples are needed to confirm this, because the steelhead trout data could not be included.

We tested the linear models by using published diet and muscle tissue FA data from salmonids to predict the muscle tissue LC-PUFA levels. The diet to muscle LC-PUFA regression models showed the strongest relationships, and when testing the model using published FA data, these relationships indicated that our models could predict the LC-PUFA in the muscle tissue based on the diet LC-PUFA level (p<0.001), especially in the case of EPA where the regression had a slope ~1 and about ¾ of the variability was explained.

### Optimal dietary FA proportions

Sufficient levels of dietary ALA should yield newly synthesized DHA in tissues. However, the proportion (or amount) of dietary ALA may not be as relevant as is its relative proportion (or amount) compared with LNA. The ratio of dietary ALA to LNA is important, as the n-3 and n-6 PUFA compete as substrates for desaturases and elongases, and subsequently for eicosanoid synthesis [34]. Excessive supplementation of ALA may not translate to higher levels of stored DHA, but rather an imbalance in the n-3 and n-6 precursors. We found that close to a 1: 1 ratio of ALA to LNA yielded the highest level of DHA stored in the muscle tissue (see Fig 2A). The ratio could also influence storage of ALA and LNA in the muscle tissue, even when DHA is supplied in minimum amounts, as suggested in the SIMPER analysis. The optimal dietary n-3 to n-6 ratio to yield ARA is lower than for DHA, at 0.6: 1 (see Fig 2B). Higher ALA to LNA ratios than the optimum resulted in lower DHA and ARA levels, and subsequently increased the proportion of ALA stored in the muscle tissue. However, the mechanisms are not totally obvious since the pathway may favor n-3 or n-6 LC-PUFA production, depending on availability and necessity.

Saturated FA in the diet were positively related to DHA in the muscle, within the range of dietary SFA inclusion that we studied (up to 40% of total FA). This relationship could relate to membrane structural requirements. For example, the phospholipid molecular species 16:0/DHA is a major contributor to phospholipid classes (including phosphatidylethanolamine, phosphatidylinositol, phosphatidylserine, and phosphatidylcholine) in marine fish and farmed trout [33], and in fact was the major contributor to the main phospholipid class (phosphatidylcholine) in trout and a majority of marine species. The positive relationship between SFA and DHA could also be due to selective catabolism of SFA in order to protect the retention of DHA. In aquaculture, this is known as the fish oil “sparing effect” [35, 36]. However, we observed a negative relationship with dietary MUFA and muscle tissue DHA, and a segmented relationship between dietary OA and DHA. Inclusion of OA up to 20% (of the total FA) results in increasing levels of DHA; higher than 20% does not result in increased storage of DHA. Our results do not suggest that OA (or total MUFA) promotes selective retention DHA.

### The tissue-to-diet concentration ratio

The tissue-to-diet concentration ratio represents the relative amount of LC-PUFA stored compared to the amount supplied in the diet. In cases where the ratio is greater than 1, it suggests sequestration and/or synthesis from n-3 or n-6 precursors, resulting in surplus LC-PUFA stored in the muscle. Where it is less than 1, disappearance is suggested. Tissue-to-diet ratios for LC-PUFA were higher in salmon than trout. This could simply reflect the size of the fish and lipid accumulation in the muscle, rather than the synthesis capability. The tissue-to-diet ratio was almost always lower in EPA than DHA and ARA, which supports our earlier evidence that suggested EPA was mobilized for other purposes. However, ratios for DHA or ARA were not consistently higher for one than the other, which may depend on conditions of the experiment, including diet formulation, species, fish size, etc. These conditions could influence n-3 or n-6 pathway synthesis. The difference in the tissue to diet ratio between diets is because of the fish oil levels. We would expect the tissue to diet ratio in fish fed the FO diet to be low because the LC-PUFA in the diet is stored directly in the tissue, thus presenting a nearly 1:1 relationship. The tissue to diet ratio in fish fed terrestrial oil-based diets show a higher ratio, meaning that when low dietary LC-PUFA is provided, synthesis is initiated, and the relative level of LC-PUFA in the muscle is greater than what is provided in the diet. Fish fed CO diets showed higher LC-PUFA tissue-to-diet values higher than FO-fed fish. Low dietary DHA increases the transcription, translation, and activity of desaturases and elongases, and as such, are under dietary influence [10, 27, 37]. Fish fed FO showed tissue-to-diet ratios at or less than 1, indicating direct storage or some level of disappearance of the LC-PUFA from consumption. For fish fed-FO, the high concentration of dietary LC-PUFA (and subsequent tissue storage) inhibits synthesis, as stimulation of the synthesis pathway reflects substrate availability [10, 27, 38]. Conversely, ALA and LNA showed tissue-to-diet ratios that were less than 1, indicating disappearance of these FA after ingestion, again due to mobilization. These results are similar to those of Emery et al. [39] who found there was little to no substrate competition between ALA and LNA when aiming at increased n-3 LC-PUFA bioconversion. The results based on our four combined studies yields new, yet general conclusions, supporting previous studies such as Emery et al. [39], that dietary LC-PUFA levels directly influence gene expression and synthesis of LC-PUFA by the fish.

## Conclusion

Strategies to improve utilization and storage of LC-PUFA in fish may include a balance of dietary n-3 to n-6 ratios, from sustainable sources. We specifically focused on the dietary ALA: LNA ratio and its effect on LC-PUFA accumulation in the muscle tissue, as looking at the total dietary n-3: n-6 is more constrained, as it does not distinguish among members of the same omega family. Abundance of ALA supply does not necessarily improve production of DHA, and while this has been generally observed in single experiments, we found that a one to one ratio of ALA to LNA optimizes DHA storage based on four experiments. Higher ALA to LNA ratios in the diet resulted in greater storage of ALA in the muscle. However, it is worth mentioning that a high n-3 to n-6 ratio (therefore a low n-6 to n-3 ratio) stored in the flesh is still beneficial for human consumption, even if a generous proportion of n-3 is supplied from ALA. A high n-6 to n-3 ratio for human consumers promotes the pathogenesis of many diseases, particularly because an inflammatory state may be activated or exacerbated by a high dietary ratio [40]. The n-6 to n-3 ratio was estimated to be 15 to 20:1 for consumers in Western societies [3], which has been linked with cardiovascular diseases [40] and cognitive decline [41]. This is further concerning if farmed seafood may not be supplying an adequate ratio of n-3 to n-6 [42]. Because consumers depend on seafood as a source of n-3 to balance high dietary n-6, balancing dietary FA inputs for farmed fish could be a beneficial strategy to optimize final muscle tissue levels of the n-3 LC-PUFA.

The conclusions based on four previous studies provided new observations toward optimizing dietary FA to improve tissue LC-PUFA profiles in salmonids. Although single experiment studies have evaluated tissue LC-PUFA content based on varying dietary FA profiles, this examination of several studies yields more general conclusions regarding dietary FA optimization. There were clearly species differences; however, there were general patterns observed that yielded new information. These new optimized dietary FA ratios should be confirmed experimentally, specifically to test the dietary ALA: LNA ratio for maximum DHA storage, and to confirm the DHA synthesis rate at ~23% using a different method.

## Supporting information

S1 Fig The linear relationship between the ALA: LNA ratio in the diet to storage of ALA in the muscle tissue of Atlantic salmon 1 (■) and 2 (▲), and rainbow trout (●). (TIF)

S2 Fig The relationship between the ALA: LNA ratio in the diet to storage of LNA in the muscle tissue of Atlantic salmon 1 (■) and 2 (▲), and rainbow trout (●) is exponential to a maximum. (TIF)

S3 Fig Segmented relationship between dietary OA and tissue DHA for Atlantic salmon 1 (■) and 2 (▲), rainbow trout (●), and steelhead trout (×), where DHA = 8.75-(0.08*OA) ≤ T; DHA = 5.40-(0.001*OA) ≥ T. (TIF)

S1 Table Results from testing linear models based on data from published literature. (PDF)

S1 File Data file for model testing. (ZIP)
