# Supplementary material for: Optimizing long chain-polyunsaturated fatty acid synthesis in salmonids by balancing dietary inputs
Source: PLoS One. 2018 Oct 10;13(10):e0205347. doi: 10.1371/journal.pone.0205347 (PMC6179257; doi:10.1371/journal.pone.0205347)
Supplement: S1 Table — (PDF) [file pone.0205347.s004.pdf]

Table S1. Results from testing linear models based on data from published literature.

Model		Slope	r^2	p-value	T-stat	p-value
Diet	Tissue					
DHA	DHA	0.74	35.5	<0.001	-0.73	0.463
EPA	EPA	1.04	73.5	<0.001	-0.60	0.549
ARA	ARA	1.02	32.3	<0.001	-2.23	0.027
ALA	DHA	1.36	13.9	<0.001	0.20	0.845
ALA	EPA	0.83	14.7	<0.001	-0.04	0.968
ALA	ARA	0.73	2.9	0.098	-2.12	0.036
LNA	DHA	0.34	14.8	<0.001	-2.37	0.019
LNA	EPA	0.17	10.1	0.001	-2.64	0.009
LNA	ARA	0.99	3.6	0.065	-1.33	0.188
SFA	DHA	0.52	12.4	0.007	-3.16	0.002
SFA	EPA	0.27	10.9	0.011	-4.89	0.000
SFA	ARA	0.51	5.5	0.091	-3.66	0.000
MUFA	DHA	0.06	0.3	0.594	-1.28	0.202
MUFA	EPA	0.25	5.2	0.028	-0.96	0.337
MUFA	ARA	0.52	2.3	0.147	-0.55	0.586
ARA	DHA	0.33	10.7	0.001	-0.69	0.488
ARA	EPA	0.56	47.8	<0.001	-1.34	0.182
ALA:LNA	DHA	0.175	1.5	0.220	7.90	0.000
ALA:LNA	ARA	0.005	0.0	0.982	2.84	0.005
